# Prevalence of Pterygium and Associated Risk Factors in the High-Altitude Area of Ta’if City, Saudi Arabia

**DOI:** 10.7759/cureus.12638

**Published:** 2021-01-11

**Authors:** Ruba Qadi, Ahmed AlAmri, Manal Elnashar, Jehan F Sarriyah, Abdulmalik H Alghamdi, Khaled Fahad Alsolami, Ashwaq M Almalki, Faisal Alotaibi

**Affiliations:** 1 Ophthalmology, Taif University, Taif, SAU; 2 Ophthalmology, King Abdulaziz Hospital, Taif, SAU; 3 Pediatrics, Taif University, Taif, SAU; 4 Medicine, Taif University, Taif, SAU; 5 Emergency Medicine, Taif University, Taif, SAU; 6 Internal Medicine, Taif University, Taif, SAU; 7 Ophthalmology, King Abdulaziz Medical City, Jeddah, SAU; 8 Ophthalmology, King Abdulaziz Specialist Hospital, Taif, SAU

**Keywords:** taif city, sunlight, ophthalmology., pterygium, high altitude

## Abstract

Background

Pterygium is an important public health problem. The prevalence rates of this disease varies widely from 1.2% to 23.4%.

Aim

To determine the prevalence rates and the associated risk factors of pterygium in the high-altitude area - Ta’if city, Saudi Arabia.

Material and method

A cross-sectional study was carried out from September 2018 till September 2019 at the ophthalmology outpatient clinics of King Abdul-Aziz Specialist Hospital, Ta’if area.

Results

Prevalence rate of pterygium in the high-altitude area, Ta’if city, Saudi Arabia was 2.4%. It is significantly higher in older patients belonging to the age group of more than 40 years. As for gender, it was significantly higher in male patients compared to females (2.6% vs. 1.9%). Pterygium prevalence was significantly higher among patients with outdoor occupations compared to indoor occupations (2.9% vs. 2.1%), and among patients with sunlight exposure during daily activities for more than 5 hours (2.6% vs. 2%) (p =< 0.05).

Conclusion

The overall incidence of pterygium in Al-Ta’if area, Saudi Arabia, was 2.4% but still lower than overall worldwide incidence (10.2%). There was an increased incidence of pterygium with age, high-altitude areas, rural areas, outdoor occupations, which is directly proportional to dose of sunlight exposure. Furthermore, smoking might be reported as a protective factor against pterygium.

## Introduction

Pterygium, a wing-shaped, fleshy growth, is an important public health problem [1]. As an invasive extraocular lesion located most commonly in the nasal part of the limbus extending onto the cornea, pterygium can trigger refractive errors as astigmatism, reduced visual acuity due to direct encroachment over the visual axis, and xerosis, which is caused by a disruption in the laminar flow of tears and conjunctival secretions [2].

Although the etiology of pterygium is unclear, the most common risk factor is ultraviolet (UV) light exposure, which induces oxidative stress and the expression of cytokines and growth factors in pterygial epithelial cells, initiating the cellular proliferation, blood vessel formation, tissue invasion, and inflammation [3]. Meanwhile, other confirmed risk factors include dry, warm, dusty climates; high winds; age; and sex [4]. Several modes of inheritance have also been reported such as autosomal-dominant, autosomal-recessive, sex-linked, and non-Mendelian modes of inheritance [5].

The prevalence rates of this disease vary widely from 1.2% to 23.4% [1], including by race, with rates of 1.2% in urban Caucasia populations and 31.01% in rural southern China reported [4]. Several studies have reported higher rates of pterygium countries near the Equator [5]. Alqahtani reported in 2013 that the prevalence rate of pterygium in Alkhobar was 0.074% [6]. A heightened prevalence rate of pterygium among populations living at high altitudes who are exposed to high levels of UVB sunlight also exists; for example, the prevalence rates of pterygium at Kathmandu (1,400 m above the sea level) was 12.4%, while that at Jharkot (3,800 m above the sea level) was 65.8% [7].

Pterygium risk factors must be modified early on since the presence of pterygia can result in significant astigmatism and visual impairment, which may affect an individual’s visual function, lifestyle, and productivity [5].

Our study sought to determine the prevalence rates and associated risk factors of pterygium in a high-altitude area - specifically Ta’if, Saudi Arabia, as Taif is a city in Mecca province at an elevation of 1,879 m (6,165 ft) on the slopes of Sarawat Mountains - for possible consideration and early modification to improve the visual function, lifestyle, and productivity.

## Materials and methods

Study design and population

This was a cross-sectional study carried out in the ophthalmology outpatient clinics of King Abdulaziz Specialist Hospital, Ta’if, Saudi Arabia from September 2018 until September 2019. Patients of all ages and both sexes who attended the selected ophthalmology clinics at the time of this study were asked to participate and those who were non-Saudis or who refused to participate were excluded. The total number of patients who attended the clinics during the study recruitment period of this study was 13,740 and, of these, 12,135 (88.3%) agreed to participate.

Study instrument

A predesigned questionnaire was administered to collect data about demographic characteristics (age, sex, residence, occupation, and smoking status). All patients were asked about their sunlight exposure during daily activities, the duration of sunlight exposure, and the use and type of eye protection during sunlight exposure. All patients were clinically evaluated by an ophthalmology specialist and the clinical information of patients diagnosed with pterygia was taken from their medical records. Clinical data included site, laterality, grade of severity, and effect of the pterygium. Data concerning previous surgical procedures for pterygia, cause of surgery, recurrence after surgery, and need for another surgery after recurrence were collected. The severity of each pterygium was determined based on its apical extent using the pen torch and a corresponding grade was awarded as follows: grade 1, the apex is at or before the limbus; grade 2, the apex is between the limbus and the pupillary margin; grade 3, the apex is at the pupillary margin; and grade 4, the apex is extended beyond the pupillary margin [8,9].

All items of the questionnaire were derived from the only national study performed in Saudi Arabia to date (in Al-Khobar) and from other international studies [6,10-12].

Ethical considerations

The Research Ethics Committee of Taif University approved the study and both written and verbal consent were obtained from the study participants.

Statistical analysis

The collected data were analyzed using the Statistical Package for the Social Sciences (SPSS) software program (IBM Corporation, Armonk, NY, USA). Numbers and percentages were used for expressing qualitative data, while the chi-squared (χ2) test was applied for testing the relationship between variables. Binary logistic regression analysis, which is a statistical tool for analyzing independent predictors with odds ratios for a binary outcome (pterygium prevalence), was performed. A statistical significance was considered to exist with a p-value of less than 0.05.

## Results

Most of the studied participants were aged 20 to 40 years (79.6%) and were male (69.1%), with rural residency (65.5%), indoor occupations (67.6%), and a current smoking habit (36.1%).

Among the study group, 81.8% of participants reported exposure to sunlight during their daily activities and 60.2% had a duration of exposure of more than five hours per day. About 52% of the participants reported using a sun-protection device such as sunglasses (79.7%) (Table 1).

**Table 1:** Descriptive data of the study participants.

Variable	n (%)
Age: <20 years, 20–40 years, >40 years	1073 (8.8), 9663 (79.6), 1399 (11.5)
Sex: Male, Female	8381 (69.1), 3754 (30.9)
Residence: Rural residence, Urban residence	7943 (65.5), 4192 (34.5)
Occupation: Outdoor occupation, Indoor occupation	3931 (32.4); 8204 (67.6)
Smoking: Smoker, Nonsmoker	4378 (36.1), 7757 (63.9)
Sunlight exposure during daily activities: Yes, No	9925 (81.8), 2210 (18.2)
Duration of sunlight exposure during daily activities: <5 hours daily, >5 hours daily	4835 (39.8), 7300 (60.2)
Use of sunlight protection device: Yes, No	6352 (52.3), 5783 (47.7)
Nature of sun protection device (n = 6352): a sunglass, a hat	5068 (79.7), 1284 (20.3)

**Figure 1:**
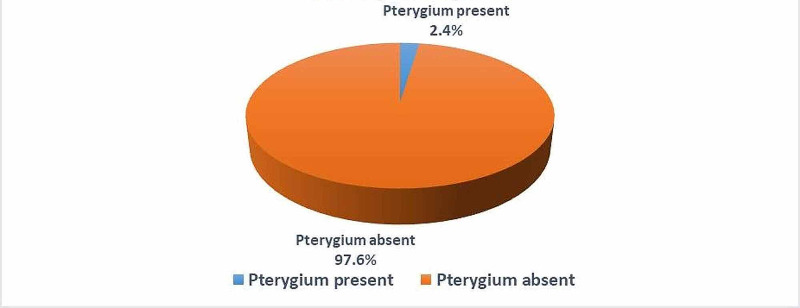
Prevalence of pterygium among the study participants.

Of the patients who attended the outpatient ophthalmology clinics at King Abdulaziz Specialist Hospital at the time of the study and accepted to participate, 289 patients were diagnosed with pterygia for a prevalence rate of 2.4% (Figure 1).

**Figure 2:**
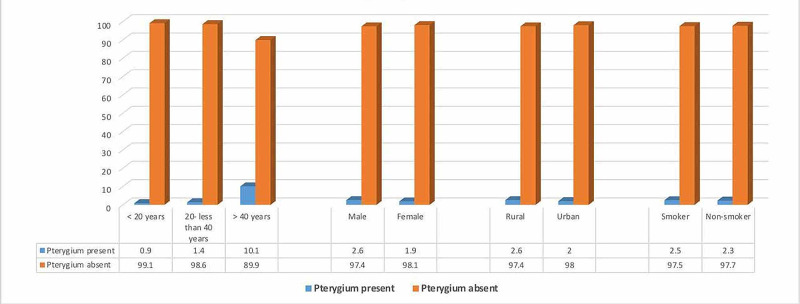
Relationship between pterygium presence and age, sex, residence, and smoking status of the study participants. For age: (χ2 = 403.99; p ≤ 0.001) For sex: (χ2 = 5.61; p = 0.018) For residence: (χ2 = 4.44; p = 0.03) For smoking: (χ2 = 0.21; p = 0.64)

Figure 2 shows that a significant relationship existed between patients with and without pterygia regarding their age, sex, and residence. Pterygium prevalence was significantly higher among patients who were older than 40 years (10.1% vs. 0.9% among those younger than 20 years and 1.4% among those 20-40 years). As for sex, pterygium prevalence was significantly higher among male patients than among female patients (2.6% vs. 1.9%). On the other hand, a nonsignificant relationship was found between patients with and without pterygia regarding smoking (p ≥ 0.05).

**Figure 3:**
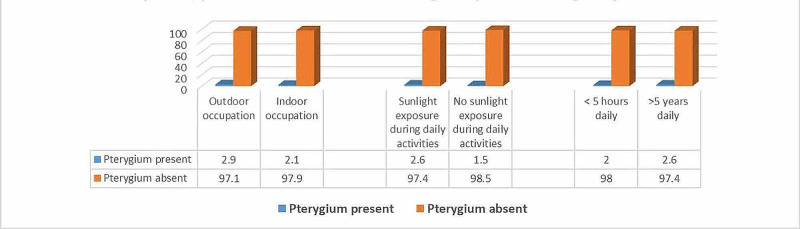
Relationship between pterygium presence and the nature of participants’ occupation and the presence and duration of sunlight exposure during daily activities. For occupation type: (χ2 = 6.72; p = 0.01) For sunlight exposure during daily activities: (χ2 = 8.26; p = 0.004) For duration of sunlight exposure: (χ2 = 4.34; p = 0.03)

Pterygium prevalence was significantly higher among patients with rural residences as compared with those living in urban areas (2.6% vs. 2%) (p ≤ 0.05). It was also significantly higher among patients with outdoor occupations as compared with indoor occupations (2.9% vs. 2.1%) and patients with sunlight exposure during daily activities lasting for more than five hours (2.6% vs. 2%) (p ≤ 0.05) (Figure 3).

Table 2 shows that, according to the clinical characteristics of the 289 patients diagnosed with pterygia, 62.6% had pterygia on the right side, 63.2% had temporal pterygia, 57% had unilateral pterygia, and 57% had grade 2 pterygia. Astigmatism and inflammation were found in about 32.6% and 57.7% of patients with pterygia, respectively. Meanwhile, 26.5% of patients underwent a surgical removal, 20.8% suffered pterygium recurrence after surgery, and 15.6% of patients with recurrent pterygia underwent another surgery for management (Table 2).

**Table 2:** Descriptive data of the clinical characteristics of patients with pterygium.

Variable	n (%)
Site of pterygium: Right, Left	171 (62.6), 102 (37.4)
Type of pterygium: Temporal, Nasal	107 (36.8), 184 (63.2)
Laterality of pterygium: Unilateral, Bilateral	166 (57), 125 (43)
Grade of pterygium: Grade 1, Grade 2, Grade 3, Grade 4	96 (33), 166 (57), 26 (8.9), 3 (1.1)
Pterygium causing astigmatism: Yes, No	95 (32.6), 196 (67.4)
Pterygium causing inflammation: Yes, No	168 (57.7), 123 (42.3)
Previous surgical removal of pterygium: Yes, No	77 (26.5), 214 (73.5)
Cause of surgery (n = 77): Cosmetic, Inflammation, Vision affected	4 (5.2), 49 (63.6), 24 (31.2)
Recurrence of pterygium after surgery (n = 77): Yes, No	16 (20.8), 61 (79.2)
Redoing the surgery after recurrence (n = 77): Yes, No	12 (15.6), 65 (84.4)

Table 3 shows that, upon conducting binary logistic regression analysis to detect the independent predictors for pterygium among studied patients, we found that being older than 40 years (male), and having a rural residence, outdoor occupation, and sunlight exposure during daily activities were independent predictors for pterygium.

**Table 3:** Binary logistic regression analysis of the risk factors for pterygium in the study population.

Variable	Pterygium prevalence			Significance
	Beta	Wald	Odd’s ratio	
Age	-1.87	252.87	0.15	0.000
Sex	2.9	4.3	1.33	0.038
Residence	3.6	7.27	1.43	0.007
Occupation type	0.28	5.05	1.32	0.025
Sunlight exposure during daily activities	0.49	6.98	1.64	0.008
Duration of sunlight exposure during daily activities	-0.21	2.75	0.8	0.09
Use of sunlight protection device	-0.029	5.74	0.74	0.017

## Discussion

The present study was conducted in a specialized hospital in Ta’if, Saudi Arabia, from 2018 to 2019. Among the total number of participants (n = 12,135), 289 were diagnosed with pterygia.

The overall prevalence of pterygium was found to be higher in Ta’if than in Al-Khobar (2.4% and 0.074%, respectively), which may be attributed to the high prevalence rate of pterygium in the populations living at higher altitudes who are exposed to high levels of UVB sunlight [7], as UV radiation can cause mutations in genes such as the P53 tumor-suppressor gene, resulting in its abnormal expression in the pterygial epithelium in a manner suggesting uncontrolled cell proliferation [13-15].

Worldwide, prevalence estimates of pterygium vary according to the study location and demographics. A meta-analysis looked at data from 20 population-based studies published in English and Chinese and found an overall prevalence of 10.2% [16]. The prevalence in Al-Khobar, Saudi Arabia was 0.074% [6], that in Iran was 1.3% [11], and that in Turkey was 3.0% [17].

Our study shows that the pterygium prevalence in older patients who were older than 40 years (10.1%) was significantly higher as compared with the prevalence rates among patients younger than 20 years and 20 to 40 years (0.9% and 1.4%). This finding is in close concordance with the findings of other studies; for example, Bueno et al. reported a significant increase in the pterygium prevalence with advancing age [18], while Elliot reported similar results in a study performed in Maoris, near the Equator [19]. In contrary with our results, however, Shrestha and Shrestha in a study in Kathmandu, Nepal showed that the prevalence of pterygium in their population was not increased with advancing in age; however, they reported that pterygium is more common among those aged 60 to 69 years (21.6%) and 70 to 79 years (23.1%) [7].

Rezvan et al. in a recent systematic review and meta-analysis study on the prevalence and risk factors of pterygium among 415,911 participants from 24 countries reported that pterygium is more prevalent among males of older ages [20]. Also, Rohatgi studied the epidemiology of pterygium in India and concluded that pterygium is more common in males than in females [21]. This correlates with our study findings where the pterygium prevalence was significantly higher among male patients than female patients (2.6% vs. 1.9%).

A recent systematic review and meta-analysis including 24 observational studies showed that cigarette smoking is inversely linked to the risk of pterygium [22]. This is in accordance with our finding where a nonsignificant relationship was observed between patients with and without pterygia regarding smoking (p ≥ 0.05). A different meta-analysis showed that cigarette smoking reduces the risk of pterygium independent of UV exposure and sex, findings which were similar to our results. Goncalves et al. explained the protective effects of smoking against pterygium could be due to the suppression of the expression of inflammatory mediators together with the vasoconstrictive effect of nicotine and cigarette smoke through stimulation of α1-adrenergic receptors followed by adrenaline and noradrenaline secretion, reducing the inflammatory response. Finally, smoking may alter components of the tear film, such as secreted antibodies [23,24].

Li et al. assessed the five-year cumulative incidence of pterygium and its associated predictors alongside two other longitudinal cohort studies and reported that outdoor occupation was the only baseline factor related to incidence of pterygium [25]. Adults performing work outdoors such as fishing or farming were 2.5 times more likely to develop pterygium in either eye as compared with those who worked indoors after adjusting for the effects of age and sex given that outdoor occupation is a proxy measure for UV exposure, which is a well-established risk factor for pterygium. This is in accordance with our results where a highly significant relationship was found among patients with pterygia and an experience of sunlight exposure during daily activities (χ2 = 8.26; p = 0.004) [25].

Di Girolamo et al. explained that UV induction of proinflammatory cytokines such as interleukin-6 and interleukin-8 might play a role in pterygium development by initiating cellular proliferation, blood vessel formation, tissue invasion, and inflammation [26]. Meanwhile, Nakagami et al. reported an increased stromal mast cells number, which led to a release of tumor necrosis factor-alpha, under UV radiation [27].

Li et al.’s study of the Bai Chinese population, who live in a rural community in China, demonstrated a relatively higher incidence of pterygium as compared with in a previous study in the Han Chinese population in an urban metropolis [25]. This is in accordance with our finding that pterygia were significantly more frequently seen among patients with rural residency as compared to those living in urban areas (2.6% vs. 2%) (p ≤ 0.05).

## Conclusions

The overall incidence of pterygium in Ta’if, Saudi Arabia was 2.4%, which is higher than that in a previous study in Al-Khobar, Saudi Arabia (0.074%) but still lower than worldwide incidence (10.2%). Regarding pterygium risk factors, an increased incidence of pterygium in those with outdoor occupations exists in a manner directly proportional to the dose of sunlight exposure received. Pterygia were more prevalent in patients living in higher-altitude areas rather than at lower altitudes and in rural areas than in urban areas. The incidence of pterygium increased with age but showed no correlation with smoking. Furthermore, smoking might actually be a protective factor against pterygium after adjusting for multiple risk factors due to the suppressed expression of inflammatory mediators.
